# Molecular and virulence characteristics of an outer membrane-associated RTX exoprotein in *Pasteurella pneumotropica*

**DOI:** 10.1186/1471-2180-11-55

**Published:** 2011-03-17

**Authors:** Hiraku Sasaki, Hiroki Ishikawa, Toru Sato, Satoshi Sekiguchi, Hiromi Amao, Eiichi Kawamoto, Tetsuya Matsumoto, Kazuhiko Shirama

**Affiliations:** 1 Animal Research Center, Tokyo Medical University, Shinjuku, Tokyo, Japan; 2 Department of Microbiology, Tokyo Medical University, Shinjuku, Tokyo, Japan; 3 Department of Histology and Neuroanatomy, Tokyo Medical University, Shinjuku, Tokyo, Japan; 4 Laboratory of Experimental Animal Science, Nippon Veterinary and Life Science University, 1-7-1 Musashino, Tokyo, Japan

## Abstract

**Background:**

*Pasteurella pneumotropica *is a ubiquitous bacterium that is frequently isolated from laboratory rodents and causes various clinical symptoms in immunodeficient animals. Currently two RTX toxins, PnxIA and PnxIIA, which are similar to hemolysin-like high-molecular-weight exoproteins are known in this species. In this study, we identified and analyzed a further RTX toxin named PnxIIIA and the corresponding type I secretion system.

**Results:**

The RTX exoprotein, PnxIIIA, contains only a few copies of the RTX repeat-like sequence and 3 large repeat sequences that are partially similar to the outer membrane protein found in several prokaryotes. Recombinant PnxIIIA protein (rPnxIIIA) was cytotoxic toward J774A.1 mouse macrophage cells, whereas cytotoxicity was attenuated by the addition of anti-CD11a monoclonal antibody. rPnxIIIA could bind to extracellular matrices (ECMs) and cause hemagglutination of sheep erythrocytes. Binding was dependent on the 3 large repeat sequences in PnxIIIA. Protein interaction analyses indicated that PnxIIIA is mainly localized in the outer membrane of *P. pneumotropica *ATCC 35149 in a self-assembled oligomeric form. PnxIIIA is less cytotoxic to J774A.1 cells than PnxIA and PnxIIA.

**Conclusions:**

The results implicate that PnxIIIA is located on the cell surface and participates in adhesion to ECMs and enhanced hemagglutination in the rodent pathogen *P. pneumotropica*.

## Background

*Pasteurella pneumotropica *is a Gram-negative rod-shaped bacterium that is frequently isolated from the upper respiratory tract of laboratory rodents. This bacterium is a major causative agent of opportunistic infection in rodents, and almost all infected immunocompetent rodents exhibit unapparent infection. An earlier study reported that coinfection by *P. pneumotropica *and *Mycoplasma pulmonis *causes pneumonia in specific pathogen-free mice [1]. A recent study reported that *P. pneumotropica *infection disturbs the inflammation responses in immunocompetent mice [2]. In immunodeficient rodents, however, *P. pneumotropica *infection leads to various serious diseases such as lethal pneumonia and sepsis. It is well known that coinfection with *Pneumocystis carinii *and *P. pneumotropica *leads to fatal pneumonia in B cell-deficient mice [3,4]. In mice lacking functional MHC II, Tlr4, and Nramp1 genes, experimental challenge with *P. pneumotropica *results in pulmonary infections [5,6]. Furthermore, orbital abscesses were caused by *P. pneumotropica *infection in Cd28-mutated mice [7]. In laboratory rodents, these infections could be effectively treated with antibiotics [8-10], and hysterotomy and embryo transfer are known to be the most effective treatments for eliminating *P. pneumotropica *completely [8]. However, both treatments are time-consuming and require special facilities and equipment. Therefore, to prevent *P. pneumotropica *infection in laboratory rodents, it is necessary to periodically perform microbiological monitoring of laboratory rodents and maintain a clean environment in the rodent colony. To perform microbiological monitoring and prevent infection, it is important to clarify the virulence factors and pathogenicity of *P. pneumotropica*.

The phenotypic characteristics related to the virulence of *P. pneumotropica *are hemagglutination and hemolysis [11-13]. Two recently named exoproteins, PnxIA and PnxIIA, both of which have C-terminal primary structures similar to the repeat in structural toxin (RTX) toxins, have been identified and characterized as hemolysin-like proteins in *P. pneumotropica *[13]. RTX toxins have many copies of glycine-rich sequences, and these toxins have been identified in many species of Gram-negative bacterium, including *Pasteurellaceae*, *Enterobacteriaceae*, and *Vibrionaceae *[14-17]. Many RTX toxins are reportedly capable of lysing erythrocytes; thus, RTX toxins function as hemolysins [14,17]. In addition, several RTX toxins act as leukotoxins and disrupt actin cytoskeletons. LtxA produced by the periodontopathogen *Aggregatibacter actinomycetemcomitans *specifically acts on human polymorphonuclear leukocytes and macrophages while concurrently lysing erythrocytes to acquire iron [18-21]. Apx toxins (ApxIA and ApxIIA) and lipopolysaccharides (LPSs) are the major virulence factors for the porcine pathogen *Actinobacillus pleuropneumoniae*, and the Apx-LPS complex promotes cytotoxicity toward porcine alveolar macrophages [22]. Furthermore, the *Vibrio cholerae *multifunctional autoprocessing RTX toxin, which acts on cellular actin protomers by cross-linking, disrupts the actin cytoskeleton of cells [23-26]. As reported in recent studies, RTX toxins act on a variety of cells and cellular matrices and are considered to have various effects on host cells. Therefore, elucidating the functions of RTX toxins may lead to a better understanding of the mechanisms by which infectious agents cause infection.

In a previous study, we identified additional members of the RTX toxin family, namely, PnxIA and PnxIIA, in *P. pneumotropica *[13]. Details about their functions and cytotoxicity, excluding their effects on sheep and mouse erythrocytes, remain to be clarified, and it is important to examine these proteins to prove that there are additional genes that code for proteins that are similar to RTX toxins; this is important for elucidating *P. pneumotropica *pathogenicity. In this study, we identified a third gene encoding an RTX protein and characterized it in terms of its *in vitro *cytotoxicity and hemolytic activity. To understand the function of this RTX protein, we attempted to determine its virulence characteristics based on its predicted primary structure.

## Results

### Identification of the third gene encoding an RTX protein

**Figure 1:**
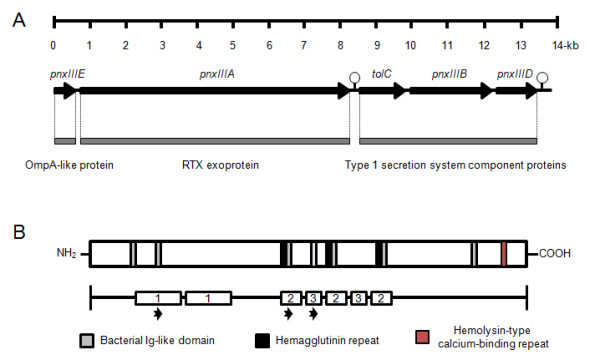
**Genetic organization and the predicted primary structure of PnxIIIA in *P. pneumotropica *ATCC 35149**. (A) Schematic representation of the *pnxIII *operon genetic map and the functions of each gene. Circles represent potential transcriptional termination loops. Predicted functions determined by the protein database are indicated below the gray boxes. (B) Schematic representation of probable domains that were identified by comparing with the HMM database. The numbers represent the regions containing a large repeat sequence. Arrowheads below the number box represent the position of sequence alignment in Additional file 1.

A previous study revealed that *P. pneumotropica *carries 2 genes encoding hemolysin-like proteins that are similar to the RTX toxins PnxIA and PnxIIA [13]. Although both structural protein-coding genes could be detected using Southern hybridization or PCR, several unspecific genes were also detected when the gene coding for PnxIIA was targeted for detection by using PCR techniques in reference strains and wild-type strains of *P. pneumotropica *(data not shown). In this study, this heterogenic PCR product was cloned, and the inserts of the resultant plasmid pTAC-PX3 were sequenced. The sequence of the inserts was similar to that of the glycine-rich regions in *pnxIIA*; however, the detailed sequence indicated the existence of an additional gene that encodes a protein similar to the RTX toxin. Subsequently, we sequenced the uninserted regions from the genomic DNA of *P. pneumotropica *ATCC 35149 by using a previously constructed clone library [13] and inverse PCR. Approximately 14 kb of related genes, including 5 putative open reading frames (ORFs), were finally identified (Figure 1A). To predict the functions of the gene products, the deduced amino acid sequence of each gene was analyzed on the basis of hidden Markov model (HMM) profiles with a protein BLAST search [27] or the Pfam database [28]. The *pnxIII *operon comprised the genes encoding 3 functional component proteins, namely, the OmpA-like protein, RTX exoprotein, and type I secretion system component proteins (Figure 1A). The deduced amino acid sequences of *tolC*, *pnxIIIB*, and *pnxIIID *were similar to that of the putative outer membrane (OM) efflux protein of *Neisseria sicca *ATCC 29256 (GenBank accession no. ZP_05317789) with 68% similarity and 91% coverage, the LapA secretion ATP-binding protein of *Neisseria mucosa *ATCC 25996 (ZP_05976520) with 86% similarity and 99% coverage, and a membrane fusion protein of *Simonsiella muelleri *ATCC 29453 (ZP_06753782) with 87% similarity and 100% coverage, respectively. The proteins from the 3 ORFs were indicated to form a type I secretion system that transports the RTX protein across the bacterial inner membrane (IM) and OM and finally exports it into extracellular space.

The *pnxIIIE *gene product contains the OmpA domain (Pfam reference: accession no. PF00691) in the C-terminus and is 54% similar to the OM protein A of *Cardiobacterium hominis *ATCC 15826 (ZP_05705729), with 84% coverage.

Although the protein BLAST search yielded no highly similar proteins, the deduced amino acid sequence of *pnxIIIA *was partially similar (46%) to the RTX family exoprotein of uropathogenic *E. coli *(UPEC) CFT073 [29] (NP_752300), i.e., 59% coverage. PnxIIIA is believed to be an essential cytotoxic protein of the structural RTX toxin.

Figure 1B shows the putative domains and repeat sequence in the primary structure of PnxIIIA. PnxIIIA did not have any significant identical conserved domains in the Pfam database; however, several partial sequences that were not significantly similar to conserved domains were identified in the HMM database. In brief, several groups of bacterial immunoglobulin (Ig)-like domains (Pfam reference: accession no. PF05345, PF02369, PF02368, PF07532, and PF10648) and a hemagglutinin repeat (PF05594) were scattered in the primary sequence of PnxIIIA, and a hemolysin-type calcium-binding repeat (PF00353) identical to nonapeptides of the RTX repeat sequence in the C-terminal half was present (Figure 1B). In particular, only 1 copy of amino acid residues in position 2319-2327 (LDGGDGNDT) was found to be identical to the RTX sequence; otherwise, 2 RTX-like sequences were found in positions 2114-2122 (*N*FGGMG*VS*N; alternate amino acid residues are italicized) and 2377-2384 (IKGGT-NDT; the missing amino acid residue is indicated with a hyphen). PnxIIIA was also found to have a unique feature: 3 regions with large repeat sequences existed, and the amino acid sequences in these regions were similar to the repeat sequences of the extracellular protein toxin identified in various prokaryotes, including important pathogens (see multiple alignments in Additional file 1). Of these, except for the unknown function of the RTX exoprotein and hemolysin-type calcium-binding protein, almost similar proteins were predicted to be localized in the OM fraction and to function as adhesive proteins.

### Cytotoxicity of rPnxIIIA

**Figure 2:**
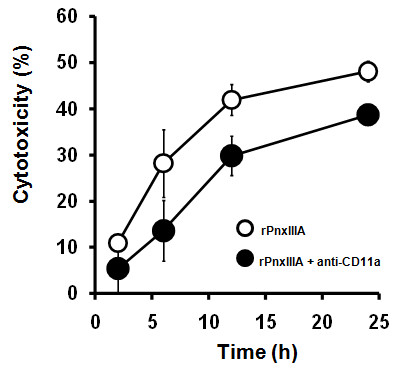
**Changes in the cytotoxicity of the rPnxIIIA toward J774A.1 mouse macrophage cells**. The cytotoxicity was determined by the release of LDH from J774A.1 cells with or without treatment with anti-CD11a monoclonal antibody cultured with rPnxIIIA.

To assess the virulence characteristics of PnxIIIA, we determined its cytotoxicity toward J774A.1 mouse macrophage cells by using soluble rPnxIIIA. With increasing rPnxIIIA concentrations, the cytotoxicity as determined from the amount of lactose dehydrogenase (LDH) released by the cells was increased during a 24-h incubation (Additional file 2). In addition, we examined and compared the cytotoxicity of 3 recombinant RTX proteins identified in *P. pneumotropica *toward J774A.1 cells. During a 4-h incubation, native rPnxIA, rPnxIIA, and rPnxIIIA exhibited 55.2% ± 7.2%, 45.2% ± 3.1% and 29.8% ± 7.1% cytotoxic to J774A.1 cells, respectively. Compared with previously found RTX proteins, rPnxIIIA was significantly less cytotoxic than rPnxIA and rPnxIIA (*P *< 0.05). Several RTX toxins have been recognized in a species-specific manner, and are found to be cytotoxic to leukocyte function-associated antigen-1 (LFA-1)-bearing cells [30-32]. To characterize the cytotoxicity of PnxIIIA toward J774A.1 mouse macrophage cells, it is important to assess the effect of the presence of the LFA-1 receptor in macrophage cells. Furthermore, we employed comparative analysis of PnxIIIA cytotoxicity by using parent J774A.1 cells and anti-CD11a monoclonal antibody (MAb)-treated J774A.1 cells as a neutralizing antibody. Figure 2 shows the changes in cytotoxicity of both J774A.1 cells and anti-CD11a MAb-treated cells cultured with 1.0 μg/ml rPnxIIIA. During a 24-h incubation, approximately 20-50% of cytolysis was inhibited by the addition of anti-CD11a MAb. These results indicate that the presence of the LFA-1 receptor may be required for rPnxIIIA cytotoxicity toward J774A.1 cells.

### ECM-binding ability and hemagglutination

**Figure 3:**
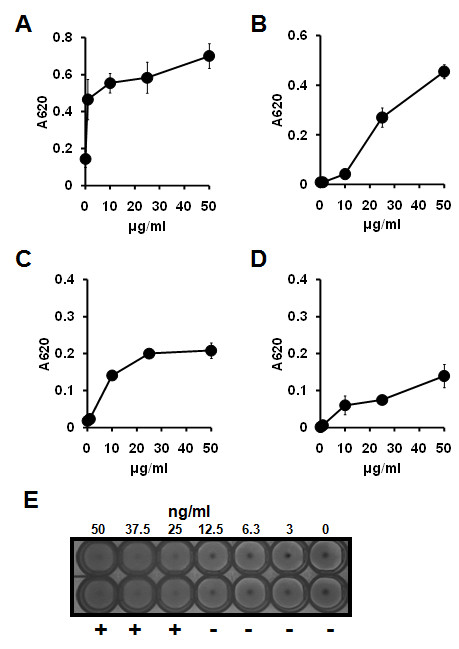
**The binding ability and hemagglutination activity of the rPnxIIIA**. The binding ability of rPnxIIIA to the ECMs as determined by ELISA (A to D) and hemagglutination activity of the rPnxIIIA with sheep erythrocytes (E). Changes in the ability of different concentrations of rPnxIIIA to bind to the rat collagen type I (A), rat collagen type II (B), mouse collagen type IV (C), and mouse laminin (D), as determined by measuring the A620. Changes in the hemagglutination activity of different concentration of rPnxIIIA with sheep erythrocytes (E).

Figures 3A to 3D show the changes in absorbance at 620 nm (A620) when rPnxIIIA was gradually added to the ECM-coated 96-well plate; the changes in absorbance were determined by an enzyme-linked immunosorbent assay (ELISA). rPnxIIIA adhered to all tested rodent ECMs, with adhesion increasing as the rPnxIIIA concentration increased. In particular, the A620 of collagen type I (Figure 3A) was highest among the tested rodent ECMs, followed by that of collagen type II (Figure 3B), which was the second most adhesive ECM at a concentration of 50 μg/ml. Although the A620 values of collagen type IV and laminin were lower than those of collagen type I and type II, rPnxIIIA was confirmed to bind to both ECMs at higher concentrations (Figure 3C and 3D). These results indicate that rPnxIIIA can bind to rodent ECMs.

When compared with the domains in the HMM database, several PnxIIIA domains have large repeat sequences that contain the hemagglutinin repeat in the primary sequence. rPnxIIIA was subjected to a hemagglutination assay with washed sheep erythrocytes. Figure 3E shows the results of the hemagglutination assay with rPnxIIIA. Hemagglutination of sheep erythrocytes was observed at rPnxIIIA concentrations exceeding 12.5 μg/ml, indicating that rPnxIIIA participates in the hemagglutination of sheep erythrocytes.

We also measured the hemoglobin released from the sheep erythrocytes when they were cultured with rPnxIIIA; however, rPnxIIIA did not exhibit typical hemolytic activity, indicating that rPnxIIIA is less involved in hemolysis.

### Characterization of deletion mutants of rPnxIIIA variants

To clarify the role of large repeat sequences in the functions of PnxIIIA, we generated soluble rPnxIIIA and deletion mutants of rPnxIIIA variants. rPnxIIIA, rPnxIIIA_209_, rPnxIIIA_197_, and rPnxIIIA_151 _essentially contained 255 kDa, 209 kDa, 197 kDa, and 151 kDa of the parent PnxIIIA, respectively (Additional file 3A).

To compare the binding ability of the rPnxIIIA variants, we performed binding assays with collagen type I coated on the 96-well plate when 10 μg/ml of the rPnxIIIA variants were applied. The A620 of wild-type rPnxIIIA was 0.55 ± 0.05, compared to 0.30 ± 0.06, 0.27 ± 0.01, and 0.26 ± 0.04 for that of rPnxIIIA_209_, rPnxIIIA_197_, and rPnxIIIA_151_, respectively (Additional file 3B). Almost all A620s of the deletion mutant proteins were lower than that of the parent rPnxIIIA. These results indicate that rPnxIIIA can bind to ECMs and that its lack of repeat sequences reduces its ability to bind ECMs.

We subjected the rPnxIIIA variants to a hemagglutination assay with washed sheep erythrocytes. Although the deletion mutant protein rPnxIII_209 _promoted hemagglutination at the same concentration as that of rPnxIIIA, more than 25 μg/ml of both rPnxIIIA_197 _and rPnxIIIA_151 _were required for hemagglutination (Additional file 3C). Although exact differentiation among the rPnxIIIA variants was not observed in hemagglutination, these results indicate that rPnxIIIA plays a role in hemagglutination and that the repeat sequences located in the C-terminal portion are necessary for enhanced hemagglutination.

### Localization and interaction of PnxIIIA

**Figure 4:**
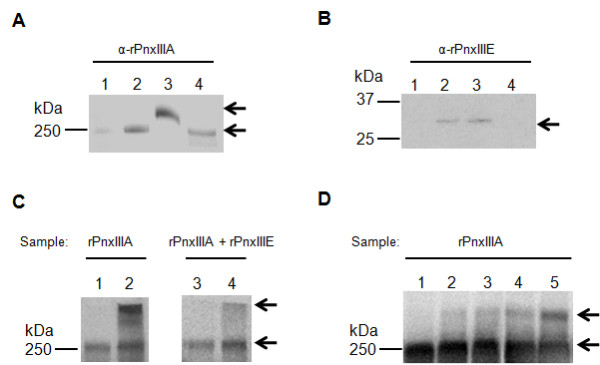
**Localization of PnxIIIA and the protein interaction analysis of rPnxIIIA**. (A) Western blotting analysis of the cell fraction prepared from *P. pneumotropica *ATCC 35149 cells and culture by using anti-rPnxIIIA IgG. Lanes: 1, SC fraction; 2, IM fraction; 3, OM fraction; 4, UC fraction. (B) Western blotting analysis of the cell fraction prepared from *P. pneumotropica *ATCC 35149 cells and culture by using anti-rPnxIIIE IgG. Lanes: 1, SC fraction; 2, IM fraction; 3, OM fraction; 4, UC fraction. (C) Western blotting analysis of rPnxIIIA by using anti-rPnxIIIA IgG after cross-linking with only rPnxIIIA or the rPnxIIIE protein and IP with anti-rPnxIIIA IgG. Lanes: 1, rPnxIIIA without cross-linking; 2, 20 μg of rPnxIIIA alone cross-linked with 50 mM BS^3 ^for 60 min and immunoprecipitated; 3, mixture of both rPnxIIIA and rPnxIIIE proteins without cross-linking; 4, 20 μg of both rPnxIIIA and rPnxIIIE proteins cross-linked with 50 mM BS^3 ^for 60 min and immunoprecipitated. (D) Western blotting analysis of rPnxIIIA by using anti-rPnxIIIA IgG after different treatment times with rPnxIIIA alone cross-linked with 50 mM BS^3 ^and immunoprecipitated with anti-rPnxIIIA IgG. Lanes: 1, rPnxIIIA without cross-linking; 2, rPnxIIIA with cross-linking for 5 min; 3, rPnxIIIA with cross-linking for 10 min; 4, rPnxIIIA with cross-linking for 30 min; 5, rPnxIIIA with cross-linking for 60 min. Arrows indicate the position of the bands that appeared.

Figure 4A shows the results of the Western blotting analysis of fractionated *P. pneumotropica *ATCC 35149 cells with anti-rPnxIIIA rabbit IgG. Signals of proteins of approximately 250 kDa in size were detected in all fractions; however, in the case of the OM fraction, the intensity of the signal was strong and located above the 250-kDa marker and other fractions. This result was also observed when the boiling time was increased to 20 min before sodium dodecyl sulfate-polyacrylamide gel electrophoresis (SDS-PAGE) and a redox reagent or denaturant such as dithiothreitol or urea was added to the OM fraction, as the position of the signal remained unchanged. Therefore, PnxIIIA appeared to tightly bind to proteins in the OM fraction. One candidate that interacts with PnxIIIA in the OM fraction is the gene product of *pnxIIIE*. Figure 4B shows the results of the Western blotting analysis of fractionated cells with anti-rPnxIIIE IgG. Signals appeared in the IM and OM fractions, and the estimated protein size was assumed to be the expected size of 30 kDa. These results may indicate that PnxIIIE exists mainly in the IM and OM fraction as a monomeric protein. Subsequently, we examined the *in vitro *interaction between rPnxIIIA and rPnxIIIE by using a soluble protein cross-linker, BS^3^. The reaction mixture was then pulled down via immunoprecipitation (IP) by using anti-rPnxIIIA IgG. Figure 4C shows the results of the Western blotting analysis of cross-linking and the IP products detected with anti-rPnxIIIA IgG. The signal was detected at 250-kDa when only rPnxIIIA or rPnxIIIA and rPnxIIIE was used alone without cross-linking (Figure 4C, lane 1 and 3). However, the positions of their signals appeared higher than that of rPnxIIIA together with the parent 250-kDa rPnxIIIA when only rPnxIIIA or rPnxIIIA and rPnxIIIE was used after treatment with 50 mM BS^3 ^(Figure 4C, lane 3 and 4). Furthermore, a shift of the signals was observed with increasing reaction time when only rPnxIIIA was used after treatment with BS^3 ^(Figure 4D). These results indicate that rPnxIIIA interacts itself, and self-assembled oligomerized PnxIIIA is located in the OM fraction in *P. pneumotropica *ATCC 35149.

**Figure 5:**
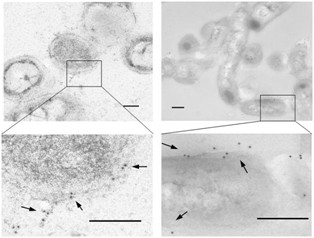
**Transmission electron micrographs of *P. pneumotropica *ATCC 35149 cells by immunoelectron microscopy with anti-rPnxIIIA IgG**. Transmission electron micrographs of the *P. pneumotropica *ATCC 35149 cells that were first reacted with anti-rPnxIIIA IgG and then labeled with gold particles (10-nm) conjugated with rabbit IgG antibody. Arrows indicate the areas where gold labeling appeared on the cell surface. Left panel, cross-section of the bacterial cell. Right panel, longitudinal section of the bacterial cell. Bar = 0.2 μm.

Figure 5 shows immunoelectron microscopy images of *P. pneumotropica *ATCC 35149 cells. Anti-rPnxIIIA IgG bound mainly to the cell surface, and few cellular and extracellular substances were gold-labeled, indicating that PnxIIIA is habitually localized on cell surfaces.

### Ability of adherence, hemagglutination, and cytotoxicity in reference strains

**Figure 6:**
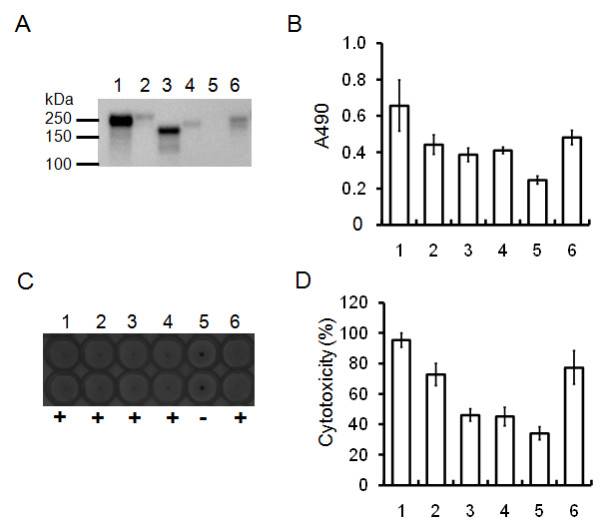
**Presence of PnxIIIA, binding ability, hemagglutination activity, and cytotoxicity of reference strains of *P. pneumotropica***. (A) Western blotting analysis of cell lysates (5 μg of total protein) of the reference strains by using anti-rPnxIIIA IgG. (B) The binding ability of the reference strains against to the rat collagen type I. A 1-way ANOVA determined that there were significant differences between the strains (*P *< 0.05). The mean value of A490 of strain ATCC 35149 (numbered as 1) or CCUG 26453 (5) is significantly different from that of the other strains by determination of Duncan's multiple-range test (*P *< 0.05). (C) Changes in hemagglutination activity of the reference strains with sheep erythrocytes. (D) Percentage of cytotoxicity determined by LDH release from the supernatant of J774A.1 cells cultured with reference strains of *P. pneumotropica*. A 1-way ANOVA determined that there were significant differences between the strains (*P *< 0.05). The mean values of cytotoxicity (%) of strain ATCC 35149 (numbered as 1) or ATCC 12555 (2) and CCUG 36632 (6) are significantly different from that of the other strains by determination of Duncan's multiple-range test (*P *< 0.05). All sections of numbers are represented as follows: 1, ATCC 35149; 2, ATCC 12555; 3, CCUG 26450; 4, CCUG 26451; 5, CCUG 26453; 6, CCUG 36632.

Initially, we performed Southern blotting analysis for detecting partial sequences of *pnxIIIA*. Only genomic DNA from *P. pneumotropica *CCUG 26450 was confirmed to include the partial gene containing the RTX repeat (Additional file 4); however, numerous signals including putative unspecific signals appeared using the probes targeting the gene encoding N-terminal portion of PnxIIIA. These results indicate that the gene encoding PnxIIIA is heterogenic and diversified. Subsequently, we performed Western blotting analysis of total protein obtained from cultured cells with anti-rPnxIIIA. Although PnxIIIA was detected in the 5 reference strains of *P. pneumotropica *by Western blotting, the estimated size and intensity of the detected signals were varied among the strains (Figure 6A). In brief, the molecular weight of the detected signals obtained from ATCC 12555 and CCUG 36632 was approximately 250 kDa, whereas those obtained from CCUG 262450 and CCUG 26451 were less than 250 kDa. Furthermore, the signals from both ATCC 35149 and CCUG 26450 had higher intensity than those of the other reference strains. The A490 values determined by whole-cell binding assays with the collagen type I of the PnxIIIA-producing strains were significantly higher than that of CCUG 26453, which was not confirmed to produce PnxIIIA (*P *< 0.05; Figure 6B). Hemagglutination activity was clearly observed in the 5 reference strains, whereas CCUG 26453 exhibits insignificant activity (Figure 6C). Although the existence of PnxIIIA was confirmed to participate in the activity of adherence and hemagglutination, these activities may be varied among the strains. Furthermore, the cytotoxicity of reference strains toward J774A.1 cells was examined (Figure 6D). Strains ATCC 35149, ATCC 12555, and CCUG 36632 exhibited more than 70% cytotoxicity; the other strains exhibited less than 50% cytotoxicity. Although the cytotoxicity of each strain did not absolutely coincide with those of the strains that produce PnxIIIA, strain CCUG 26453, which was not confirmed to produce PnxIIIA, was demonstrated to be less cytotoxic toward J774A.1 cells. These results also indicate that rodent isolates were found to have binding and hemagglutination activities; on the other hand, *P. pneumotropica *CCUG 26453, which was recorded to be isolated from birds, was not confirmed to have these activities (Table 1).

**Table 1:** Bacterial strains and plasmids used in this study

Strain or plasmid	Description	Source or reference
Strains		
*Pasteurella pneumotropica*		
ATCC 35149	Type strain, biotype Jawetz, isolated from mouse lung	ATCC^a ^[50]
ATCC 12555	Biotype Heyl, isolated from mouse	ATCC [51]
CCUG 26450	Biotype Jawetz, isolated from gerbil	CCUG^b^
CCUG 26451	Biotype Jawetz, isolated from hamster	CCUG
CCUG 26453	Biotype Heyl, isolated from bird	CCUG
CCUG 36632	Biotype unknown, isolated from murine nose	CCUG
*Escherichia coli*		
DH5α	Cloning strain	Stratagene
TOP10	Cloning strain	Invitrogen
BL21-AI	Protein expression strain	Invitrogen
TMU0812	BL21-AI Δ*hlyE*::Km^r^	[13]
		
Plasmids		
pTAC-1	Cloning vector, Ap^r^	Biodynamics Laboratory
pENTR/SD/D-TOPO	Entry vector, Km^r^	Invitrogen
pBAD-DEST49	Protein expression vector, N-terminal fusions to thioredoxin tag and C-terminal fusions to six-Histidine tag, Ap^r^	Invitrogen
pET300/NT-DEST	Protein expression vector, N-terminal fusions to six-Histidine tag, Ap^r^	Invitrogen
pTAC-PX3	0.5-kb *pnxIIIA *PCR fragment	This study
pBAD-Pnx3A	Entire *pnxIIIA *gene cloned into pBAD-DEST49	This study
pBAD-Pnx3A_209_	1.3-kb sequence of repeat 1 deleted from pBAD-Pnx3A	This study
pBAD-Pnx3A_197_	1.7-kb sequence of repeats 2 and 3 deleted from pBAD-Pnx3A	This study
pBAD-Pnx3A_151_	3.0-kb sequence of repeats 1, 2, and 3 deleted from pBAD-Pnx3A	This study
pET-Pnx3E	Entire *pnxIIIE *gene cloned into pET300/NT-DEST	This study

^a^American Type Culture Collection

^b^Culture Collection of the University of Göteborg

## Discussion

In this study, we identified and characterized a third gene that encodes an RTX exoprotein in *P. pneumotropica*. A known protein that is similar to PnxIIIA is the RTX exoprotein, which was identified in a UPEC strain [29]. Lloyd et al. [33] reported that a mutant strain in which the gene encoding this RTX exoprotein was deleted colonized bladders and kidneys less efficiently than the wild-type UPEC strain. These results indicate that this RTX toxin may participate in bacterial colonization. To characterize the virulence properties of PnxIIIA, we focused on its adhesion and hemagglutination activities as well as its cytotoxicity. For instance, 100-500 ng/ml recombinant CyaA from *Bordetella pertussis *lysed approximately 100% of murine monocytes over a 4-h period [34]. Although the conditions were different, PnxIIIA was assumed to be weakly cytotoxic compared to the RTX toxin, which is highly toxic.

Several RTX toxins that act as leukotoxins reportedly bind to β_2_-integrin LFA-1 (CD11a/CD18) on species-specific leukocytes [30-32,35]. LFA-1 is expressed on the cell surface as a glycoprotein composed of the α subunit of CD11 and the β subunit of CD18. In the case of LktA produced by *Mannheimia haemolytica*, which is the principal pathogen of bovine respiratory diseases complex, can bind to the bovine CD11a of LFA-1 [31]. LtxA produced by *A. actinomycetemcomitans *recognizes the β-propeller domain of human CD11a [36]. The cytotoxicity of rPnxIIIA toward J774A.1 cells was successfully attenuated by the addition of anti-CD11a MAb, which can react to mouse CD11a as a neutralizing antibody, suggesting that the α subunit of mouse LFA-1 may be required for its cytotoxicity toward J774A.1 cells. The detailed mechanisms underlying CD11a mediated PnxIIIA cytolysis need to be clarified in future studies.

One of the features of this high-molecular-weight protein is that it has 2-3 different copies of 3 large repeat sequences. These copies, although not completely identical, are highly similar and contain several bacterial Ig-like domains and a hemagglutination repeat. The deletion mutant proteins were observed to bind less to rodent ECMs compared with the parent rPnxIIIA. All 3 large repeat sequences contained regions that were partially similar to several groups of bacterial Ig-like domains, including groups 1, 2, and 4. Many Ig-like domains that belong to these groups are indicated to form an Ig-like fold and are reportedly present in bacterial cell-surface proteins such as intimins and invasins [37-40]. The other groups of Ig-like domains have also been suggested to form Ig-like folds and play in a role in adhesion to host cells, contributing to pathogenicity [41]. In accordance with our experimental results, these sequences are indispensable for adherence to ECMs, and thus, the 3 large repeat sequences in PnxIIIA may be required for the pathogenicity of *P. pneumotropica*.

All RTX proteins in *P. pneumotropica *have only 3-7 RTX repeats and RTX-like sequences, and the numbers of the repeat sequence are fewer than those in the other highly toxic members of RTX toxin family [15,17]. For example, the toxicity of the *B. pertussis *RTX toxin CyaA is reportedly activated by the coexpression of its accessory protein acyltransferase CyaC, leading to the binding of *B. pertussis *to eukaryotic cells [42,43]. In the 3 RTX toxins in *P. pneumotropica*, none of the predicted acylation protein-coding genes were found in neighboring genes, and the acylation site was also not found in the primary structure of the proteins, indicating that the RTX proteins identified in *P. pneumotropica *have a structure that is unique to the RTX toxin family. Furthermore, the phenotypic and genetic characteristics of wild-type strain of *P. pneumotropica *were reportedly diversified with an increase in the number of isolates [44]. PnxIIIA is also assumed to be heterogenic and diversified among the *P. pneumotropica *strains. It is necessary to further clarify the relationships between the diversity and the role of PnxIIIA in *P. pneumotropica *infection.

## Conclusions

In this study, we identified and characterized a third gene encoding the RTX exoprotein PnxIIIA. The results indicated that rPnxIIIA has cytotoxicity toward J774A.1 cells. Our results also implicate that PnxIIIA is localized on the cell surface and is related to adherence to the host ECMs and hemagglutination.

## Methods

### Bacterial strains and plasmids

The *P. pneumotropica *reference and *E. coli *strains and plasmids used in this study are listed in Table 1.

*pnxIIIA *was first amplified using the primer pair pnx3A-pcr-f and pnx3A-pcr-r (Additional file 5 lists the oligonucleotide primers), and subsequently, the purified PCR product was used for a second amplification of *pnxIIIA *by using the primer pair pnx3A-protein-f and pnx3A-protein-r. The amplicon was cloned into an entry vector, pENTR/SD/D-TOPO vector (Invitrogen, Carlsbad, CA, USA), and subsequently recombined with the destination vector pBAD-DEST49 (Invitrogen), yielding pBAD-Pnx3A. Mutant PnxIIIA expression vectors, pBAD-Pnx3A_209_, pBAD-Pnx3A_197_, and pBAD-Pnx3A_151_, were also constructed as described below.

### Bacterial and cell cultures and growth conditions

All *P. pneumotropica *strains were maintained in a brain-heart infusion medium (BD, Cockeysville, MD, USA) at 37°C and incubated for 48 h. Transformed *E. coli *bacteria were grown at 37°C for 16 h in Luria-Bertani medium supplemented with 100 μg/ml ampicillin, 50 μg/ml kanamycin, 125 μM 5-bromo-4-chloro-3-indolyl-β-D-galactopyranoside, and 1 mM isopropyl-β-D-thiogalactopyranoside, if required, to select and maintain recombinant *E. coli*. To induce gene expression in the recombinant *E. coli*, the cells were incubated at 37°C for 2-3 h until the optical density (OD, 600 nm) reached 1.0. Subsequently, 0.1% L-arabinose was added to the culture. During the induction of gene expression, the cell culture was incubated at room temperature (RT) for 16 h.

J774A.1 mouse macrophage cells (JCRB9108) were provided by Health Science Research Resources Bank (Osaka, Japan). The J774A.1 cells were cultivated at 37°C in 5% CO_2 _in Dulbecco's modified Eagle medium (DMEM; Wako, Osaka, Japan) supplemented with 10% fetal bovine serum, 100 U penicillin, and 100 μg/ml streptomycin sulfate.

### Nucleic acid extraction and purification

Plasmid and genomic DNA were extracted according to the method described in a previous study [45].

### TA cloning, inverse PCR, and DNA sequencing

A fragment of *pnxIIIA *was amplified with the primer pair pnx2A-f and pnx2A-r by using Ex *Taq *(Takara Bio, Shiga, Japan), and the amplified product was purified using SUPREC-PCR (Takara Bio). The purified PCR amplicons were ligated with the pTAC-1 vector (Biodynamics Laboratory, Tokyo, Japan), and *E. coli *DH5α was transformed with the resultant vectors. The clones were screened via blue-white selection and direct colony PCR by using the M13 primer pair. For inverse PCR, the genomic DNA of *P. pneumotropica *ATCC 35149 was digested with various restriction enzymes that recognized a 6-nucleotide sequence, and subsequently, the digestion product was self-ligated with T4 ligase (Takara Bio) and then used as an inverse PCR template. Inverse PCR was performed using gradient PCR to determine the optimum annealing temperature for a model DNA Engine PTC-200 (Bio-Rad Laboratories, Hercules, CA, USA). The PCR products were ligated with the pTAC-1 vector and screened to ensure the accuracy of sequencing. Cycle sequencing was performed using the BigDye terminator premix (Applied Biosystems, Foster City, CA, USA). The products of the sequencing reaction were analyzed using an ABI 310 or ABI 3730XL DNA analyzer (Applied Biosystems).

### Purification of recombinant Pnx proteins

rPnxIIIA was extracted and purified from the cell culture of *E. coli *strain TMU0812 harboring pBAD-Pnx3A. The cultured cells were suspended in 20 mM Tris-HCl, 150 mM NaCl, 5 mM imidazole, and 1 mM 2-mercaptoethanol (pH 8.0, binding buffer); they were then broken by sonication. The sonicate was centrifuged at 7,000 × *g *for 10 min and filtered using a 0.45-μm filter unit (Millipore, Billerica, MA, USA). The supernatant was loaded onto a 1-ml His-trap HP affinity column (GE Healthcare, Amersham, UK) mounted on an ÁKTAprime plus fast protein liquid chromatography device (FPLC device; GE Healthcare), and chromatography was performed by running a program for histidine-tagged protein purification according to the manufacturer's instructions. The collected sample was then eluted as a fraction in a buffer containing 20 mM Tris-HCl, 150 mM NaCl, 500 mM imidazole, and 1 mM 2-mercaptoethanol (pH 8.0; elution buffer). Fractions that mainly contained rPnxIIIA were monitored and confirmed by SDS-PAGE. For purification of rPnxIIIE, *E. coli *BL21-AI cultures harboring pET-Pnx3E were extracted in a binding buffer containing 6 M guanidine hydrochloride, and the extracts were purified with an elution buffer containing 6 M urea, similar to the method used to purify rPnxIIIA. The solvent of rPnxIIIA and rPnxIIIE was exchanged to a buffer containing 20 mM Tris-HCl and 150 mM NaCl by using FPLC and dialysis, respectively.

Purification of native rPnxIA and rPnxIIA was performed briefly according to previous described methods [13].

### Generation of deletion mutants of rPnxIIIA variants

To compare the function of the unique repeat sequences in the rPnxIIIA variants, deletion mutant rPnxIIIA expression vectors were constructed. In brief, deletion mutant expression vectors pBAD-Pnx3A_209_, which lacked amino acid residues of a repeat sequence at position 287-735 (Figure 1B; Repeat 1), and pBAD-Pnx3A_197_, which lacked amino acid residues of a repeat sequence at position 1097-1666, (Figure 1B; Repeats 2 and 3) were directly constructed using the wild-type protein expression vector pBAD-Pnx3A as the template with primer pairs pnx3A-209-f and pnx3A-209-r and pnx3A-197-f and pnx3A-197-r, respectively. A PrimeSTAR Mutagenesis Basal Kit (Takara Bio) was used to create these deletion mutant expression vectors. Finally, pBAD-Pnx3A_151_, which lacked both repeat sequences, was constructed with the primer pair pnx3A-197-f and pnx3A-197-r with pBAD-Pnx3A_209 _as the PCR template. All the constructs were confirmed with DNA sequencing. The expression and purification of rPnxIIIA variants were performed in the same manner as that used for the wild-type rPnxIIIA.

### Cytotoxicity assay

The cytotoxicity of the recombinant Pnx proteins toward J774A.1 cells was determined via a LDH release assay that was performed according to the methods of Basler et al. [34] with minor modifications. Prior to incubation, the concentration of J774A.1 cells in a 96-well plate was adjusted 1 × 10^5 ^cells per well. The cells were grown in fresh DMEM supplemented with 20 mM CaCl_2 _and appropriate antibiotics. rPnxIIIA was added to the wells such that its concentrations were 0.1, 0.5, and 1.0 μg/ml of the final concentrations. The plate was incubated at 37°C in 5% CO_2 _for up to 24 h. LDH release from the J774A.1 cells was measured at 1, 2, 4, 6, 12, and 24 h by using the supernatant from the treated cells; a cytotoxicity detection kit (Roche Diagnostics, Mannheim, Germany) was used for this purpose. For the comparison of cytotoxicity among rPnxIA, rPnxIIA, and rPnxIIIA, 1.0 μg/ml of each recombinant protein was incubated with the J774A.1 cells for 4 h. Thereafter, LDH release from the J774A.1 cells was measured. Furthermore, to assess the effect of existence of CD11a on inhibition of rPnxIIIA-induced cytolysis, LDH release from the J774A.1 cells with the addition of 1.0 μg/ml rPnxIIIA with or without 1.0 μg/ml anti-CD11a rat MAb (Abcam, Cambridge, UK), which cross-reacts with the α subunit of mouse CD11 as a neutralizing antibody, was measured after 2, 6, 12, and 24 h of incubation as described above. To assess the cytotoxicity of *P. pneumotropica *reference strains toward J774A.1 cells, a whole bacterial cell cytotoxicity assay was performed briefly according to the methods of Kehl-Fie et al. [46]. The results were reported as the percentage of LDH released from all the lysed cells. The experiments were repeated in triplicate.

### ECM-binding assay

ELISA was used to determine the binding of rPnxIIIA variants to components of rodent ECMs. In brief, a 96-well microtiter plate coated with rat collagen type I (BD BioCoat, BD) was used for the binding assay of rat collagen type I, and rat collagen type II (Chondrex, Redmond, WA, USA), mouse collagen type IV (BD), and mouse laminin (BD) were differently coated on a 96-well microplate (As one, Osaka, Japan) according to the manufacturer's instructions. ELISA was performed with a protein detector AP microwell kit (KPL, Gaithersburg, MD, USA), anti-6× Histidine tag monoclonal antibody (Biodynamics laboratory), and rPnxIIIA variants, the concentrations of which were adjusted to 0.5-50 μg/ml of the final concentration.

For the whole-cell binding assay of *P. pneumotropica *reference strains, precultured cells were adjusted the OD reached 1.0 and incubated on a 96-well microtiter plate coated with rat collagen type I (BD) at 37°C for 24 h. Thereafter, the plate was briefly washed and stained with 0.1% safranin, according to the method of Davey and Duncan [47]. The quantification of adherent cells was determined by measuring the A490 with a plate reader.

### Hemagglutination and hemolytic assay

Defibrinated sheep blood was obtained from Nippon Bio-Test Laboratories (Tokyo, Japan) and washed 3 times with sterilized phosphate-buffered saline (PBS) prior to conducting the assays. Hemagglutination activity was determined in V-cut bottom 96-well microtiter plates (Corning, Horseheads, NY, USA). Fifty micro milliliters of diluted rPnxIIIA variants or overnight cultures of *P. pneumotropica *reference strains were added to wells containing 2% sheep erythrocytes. The plate was incubated at RT for 1 h, and thereafter, the plate was imaged and visualized with a GeneGenius Bio Imaging System (Syngene, MD, USA). A hemolysis assay was performed according to a previously described method [13] that used 2% sheep erythrocytes.

### Generation and purification of rabbit antisera

In brief, crude rabbit antisera against the entire rPnxIIIA and rPnxIIIE proteins were prepared using the methods of Schaller et al. [48]. The crude antisera were further purified on an HiTrap rProtein A FF column (GE Healthcare) mounted on an FPLC device, and rabbit IgG was prepared for immunological experiments. The Institutional Animal Care and Use Committee of Tokyo Medical University approved all of the animal experimental procedures described here.

### Fractionation of bacterial cell culture

Fractionation of the OM fraction, IM fraction, and soluble cell (SC) components was performed according to the methods of Valle et al. [49]. *P. pneumotropica *ATCC 35149 cells in the mid-log phase were harvested, resuspended in 10 mM HEPES (pH 7.5) with 50 mM NaCl and 0.1 mg/ml lysozyme, and disrupted by sonication. The sonicate was centrifuged at 7,000 × *g *for 10 min, and subsequently, the supernatant was centrifuged at 100,000 × *g *for 1 h by using a Beckman Optima TL Tabletop Centrifuge (Beckman Coulter, Brea, CA, USA). The supernatant was used as the SC fraction, and the pellet containing the bacterial membrane was resuspended in a buffer containing 0.5% sarkosyl (*N*-laurylsarcosine) and allowed to stand for 30 min at RT. The sarkosyl-soluble fraction was centrifuged at 100,000 × *g *for 1 h. The supernatant was used as the IM fraction, and the pellet was resuspended in a 500 μl of 10 mM HEPES (pH 7.5) with 50 mM NaCl, 1% sarkosyl, and 10 mM EDTA and used as the OM fraction. To prepare a cell-free supernatant, the *P. pneumotropica *ATCC 35149 culture in the mid-log phase was centrifuged at 7,000 × g for 10 min, and the supernatant was filtered through a 0.22-μm pore size filter (Millipore) followed by a 0.45-μm pore size filter (Millipore). The filtrate was ultrafiltrated at 1000 × *g *for 20 min by using AmiconUltra-15 (Millipore). The resultant solution was used as the ultrafiltrated culture supernatant (UC) fraction. For SDS-PAGE analysis, the concentration of the SC, IM, OM, and UC samples were adjusted to 0.2 mg/ml, and 10 μl of each sample were subjected to 10% SDS-PAGE.

### Cross-linking and pull-down assay

To determine the *in vitro *interaction of rPnxIIIA and rPnxIIIE, chemical cross-linking and IP were performed. A cross-linker for soluble proteins, bis[sulfosuccinimidyl] suberate-d_0 _(BS^3^-d_0_; Thermo Fisher Scientific, Waltham, MA, USA), was used for the cross-linking reaction of rPnxIIIA and rPnxIIIE according to the manufacturer's instructions. To terminate the cross-linking reaction, 20 mM NH_4_HCO_3 _was added. Thereafter, a mixed solution was subjected to IP by using an IP kit, Dynabeads Protein G (Invitrogen), and rabbit IgG against rPnxIIIA according to the manufacturer's instructions. The resultant solution was subjected to SDS-PAGE, and the interaction of rPnxIIIA with rPnxIIIA or rPnxIIIE was detected by Western blotting as described below.

### Western blotting and Southern hybridization

Fractions of the *P. pneumotropica *cell culture, IP-treated sample, and cell lysates of *P. pneumotropica *reference strains were analyzed by Western blotting by using anti-rPnxIIIA IgG (1:20,000) or anti-rPnxIIIE IgG (1:20,000), followed by SDS-PAGE separation. Anti-rabbit IgG antibody conjugated to horseradish peroxidase (HRP; Thermo Fisher Scientific) for anti-rPnxIIIA IgG was used as secondary antibodies at a dilution of 1:50,000. To detect hybridization signals, the Femto Western chemiluminescence reagent (Thermo Fisher Scientific) was used as a substrate for HRP.

For Southern hybridization, digoxigenin-11-dUTP-labeled *pnxIIIA *probes were generated using the primer-pair pnx3A-probe-f and pnx3A-probe-r and the genomic DNA of *P. pneumotropica *ATCC 35149. The genomic DNAs of the reference strains were digested with *Hin*dIII and loaded on agarose gels. The hybridization and detection protocol used has been described previously [13].

### Immunoelectron microscopy

Bacterial cells were fixed with 4% (w/v) paraformaldehyde, 0.25% (v/v) glutaraldehyde, and 5% sucrose in 1.5 ml of 0.1 M phosphate buffer (pH 7.4) for 2 h at 4°C. The cells were harvested at 1000 × *g *for 10 min. The pellets were then rinsed with the same buffer and dehydrated by passing them through an ethanol series. Samples were embedded in LR-white resin. Thin sections were placed in PBS with 5% bovine serum albumin (BSA) for 30 min at RT and then incubated with rabbit anti-PnxIIIA IgG diluted to 1:100 with 1% BSA in PBS for 4 h at RT. The sections were washed 3 times in PBS and incubated with goat anti-rabbit IgG conjugated with 10-nm immunogold particles (BBInternational, Cardiff, UK) diluted to 1:50 with 5% BSA in PBS for 1 h. The sections were subsequently stained with uranyl acetate and lead citrate and viewed under a JEOL JEM-1200EX electron microscope (JEOL, Tokyo, Japan) at 80 kV.

### Nucleic acid accession numbers

The nucleotide sequences of *pnxIIIE*, *pnxIIIA*, *pnxIIIB*, *pnxIIID*, and *tolC *were deposited in GenBank through DNA Data Bank of Japan, and the assigned accession numbers were AB568084, AB568085, AB568086, AB568087, and AB568088, respectively.

## Authors' contributions

HS performed all the examinations and coordinated the study. HI and TM supervised the experimental conditions. TS, KK, and KS analyzed immunoelectron microscopy data and supported the study. SS and HA performed the purification of recombinant proteins and cytotoxicity assays. All authors read and approved the final manuscript.

## Supplementary Material

Additional file 1 **Multiple alignments of the 3 regions with repeat sequences in PnxIIIA**. The numbers at the terminus represent the position of each protein. Identical residues and similarity substitutions are highlighted in black and gray, respectively. Each organism and protein are represented by abbreviations as follows: PN, PnxIIIA from *P. pneumotropica *ATCC 35149; PR, RTX family exoprotein A from *Proteus mirabilis *ATCC 29906 (accession no., EEI46927); EC, putative RTX family exoprotein from *E. coli *CFT073 (AAN78844); CA, cell wall surface anchor family protein from *Cardiobacterium hominis *ATCC 15826 (EEV87836); AN, possible LPXTG anchored adhesin from *Anaerococcus tetradius *ATCC 35098 (EEI83830); MH, hemolysin-type calcium-binding protein from *Marinomonas *sp. strain MWYL1 (ABR70778); VC, RTX toxin from *V. cholerae *M66-2 (ACP05873); PS, putative outer membrane adhesin-like protein from *Psychrobacter *sp. PRwf-1 (ABQ94037); FL, probable aggregation factor core protein MAFp3 from *Dokdonia donghaensis *MED134 (EAQ39910); ST, putative RTX family exoprotein from *Streptococcus suis *98HAH33 (ABP91341). Click here for file

Additional file 2 **Changes in cytotoxicity toward J774A.1 cells cultured with different concentrations of rPnxIIIA**. The cytotoxicity was determined by the release of LDH from J774A.1 mouse macrophage cells. Click here for file

Additional file 3 **The binding ability and hemagglutination activity of the rPnxIIIA variants**. (A) Coomassie blue-stained SDS-PAGE analysis of rPnxIIIA variants. Lanes: M, protein ladder; 1, wild-type rPnxIIIA; 2, rPnxIIIA_209_; 3, rPnxIIIA_197_; 4, rPnxIIIA_151_. (B) Ability of rPnxIIIA variants (10 μg/ml) to bind to the rat collagen type I measured by A620. Numbers are represented as follows: 1, wild-type rPnxIIIA; 2, rPnxIIIA_209_; 3, rPnxIIIA_197_; 4, rPnxIIIA_151_. (C) Changes in hemagglutination activity of different concentration of the rPnxIIIA variants with sheep erythrocytes. Numbers are represented as follows: 1, rPnxIIIA_209_; 2, rPnxIIIA_197_; and 3, rPnxIIIA_151_. Click here for file

Additional file 4 **Southern blotting analysis of reference strains of *P. pneumotropica *using *pnxIIIA *probes**. The arrow indicates the position of the expected bands. Click here for file

Additional file 5 **Oligonucleotide primers used in this study**. Primer name, sequence, target gene, and their purpose are listed. Click here for file
