# Supplementary figures and images for: Molecular and virulence characteristics of an outer membrane-associated RTX exoprotein in Pasteurella pneumotropica
Source: BMC Microbiol. 2011 Mar 17;11:55. doi: 10.1186/1471-2180-11-55 (PMC3075217; doi:10.1186/1471-2180-11-55)

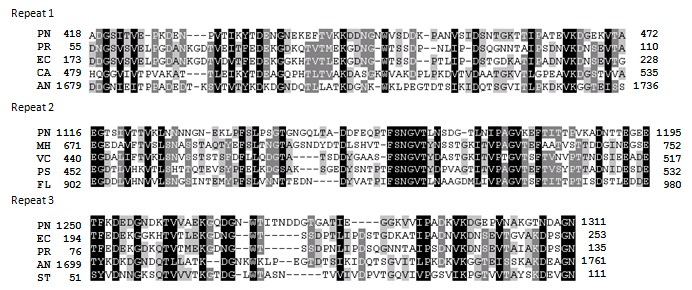

Supplement: Additional file 1 — Multiple alignments of the 3 regions with repeat sequences in PnxIIIA. The numbers at the terminus represent the position of each protein. Identical residues and similarity substitutions are highlighted in black and gray, respectively. Each organism and protein are represented by abbreviations as follows: PN, PnxIIIA from P. pneumotropica ATCC 35149; PR, RTX family exoprotein A from Proteus mirabilis ATCC 29906 (accession no., EEI46927); EC, putative RTX family exoprotein from E. coli CFT073 (AAN78844); CA, cell wall surface anchor family protein from Cardiobacterium hominis ATCC 15826 (EEV87836); AN, possible LPXTG anchored adhesin from Anaerococcus tetradius ATCC 35098 (EEI83830); MH, hemolysin-type calcium-binding protein from Marinomonas sp. strain MWYL1 (ABR70778); VC, RTX toxin from V. cholerae M66-2 (ACP05873); PS, putative outer membrane adhesin-like protein from Psychrobacter sp. PRwf-1 (ABQ94037); FL, probable aggregation factor core protein MAFp3 from Dokdonia donghaensis MED134 (EAQ39910); ST, putative RTX family exoprotein from Streptococcus suis 98HAH33 (ABP91341). [file 1471-2180-11-55-S1.BMP]

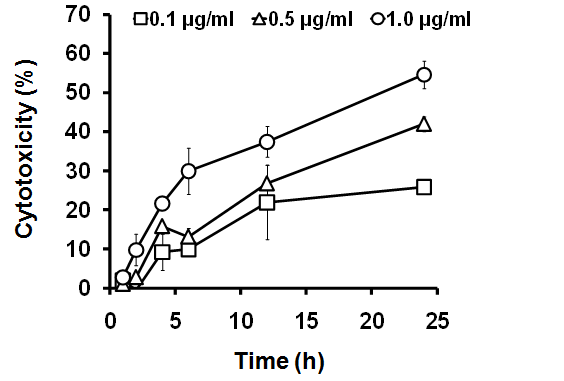

Supplement: Additional file 2 — Changes in cytotoxicity toward J774A.1 cells cultured with different concentrations of rPnxIIIA. The cytotoxicity was determined by the release of LDH from J774A.1 mouse macrophage cells. [file 1471-2180-11-55-S2.BMP]

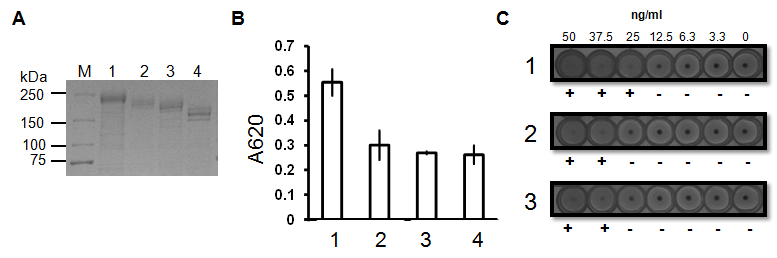

Supplement: Additional file 3 — The binding ability and hemagglutination activity of the rPnxIIIA variants. (A) Coomassie blue-stained SDS-PAGE analysis of rPnxIIIA variants. Lanes: M, protein ladder; 1, wild-type rPnxIIIA; 2, rPnxIIIA209; 3, rPnxIIIA197; 4, rPnxIIIA151. (B) Ability of rPnxIIIA variants (10 μg/ml) to bind to the rat collagen type I measured by A620. Numbers are represented as follows: 1, wild-type rPnxIIIA; 2, rPnxIIIA209; 3, rPnxIIIA197; 4, rPnxIIIA151. (C) Changes in hemagglutination activity of different concentration of the rPnxIIIA variants with sheep erythrocytes. Numbers are represented as follows: 1, rPnxIIIA209; 2, rPnxIIIA197; and 3, rPnxIIIA151. [file 1471-2180-11-55-S3.BMP]

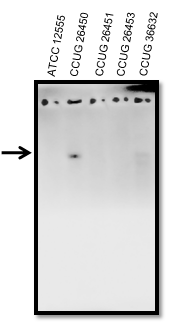

Supplement: Additional file 4 — Southern blotting analysis of reference strains of P. pneumotropica using pnxIIIA probes. The arrow indicates the position of the expected bands. [file 1471-2180-11-55-S4.BMP]

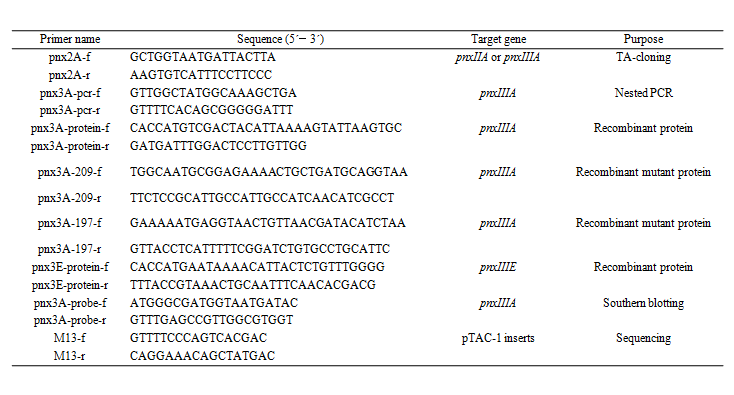

Supplement: Additional file 5 — Oligonucleotide primers used in this study. Primer name, sequence, target gene, and their purpose are listed. [file 1471-2180-11-55-S5.BMP]
